# Seroepidemiology of Severe Acute Respiratory Syndrome Coronavirus 2 Infection in Blood Donors from Western Romania, August–September 2023

**DOI:** 10.3390/microorganisms13102313

**Published:** 2025-10-06

**Authors:** Tudor Rares Olariu, Rodica Lighezan, Sorin Ursoniu, Alina Cristiana Craciun, Alexander Tudor Olariu, Sergiu Adrian Sprintar, Daniela Adriana Oatis, Maria Alina Lupu, Alin Gabriel Mihu

**Affiliations:** 1 Discipline of Parasitology, Department of Infectious Diseases, Victor Babes University of Medicine and Pharmacy, 300041 Timisoara, Romania; lighezan.rodica@umft.ro (R.L.); lupu.alina@umft.ro (M.A.L.); 2 Clinical Laboratory, Municipal Clinical Emergency Hospital, 300254 Timisoara, Romania; alinacristianacraciun@yahoo.com; 3 Center for Diagnosis and Study of Parasitic Diseases, Department of Infectious Disease, Victor Babes University of Medicine and Pharmacy, 300041 Timisoara, Romania; sergiu.sprintar@umft.ro (S.A.S.); alin.mihu@umft.ro (A.G.M.); 4 Patogen Preventia, 300124 Timisoara, Romania; alexanderolariu@yahoo.com; 5 Regional Blood Transfusion Center, 300737 Timisoara, Romania; 6 Department of Functional Sciences, Discipline of Public Health, Center for Translational Research and Systems Medicine, Victor Babes University of Medicine and Pharmacy, 300041 Timisoara, Romania; sursoniu@umft.ro; 7 Department of General Medicine, Vasile Goldiș Western University of Arad, 310048 Arad, Romania; 8 Department of Biology and Life Sciences, Faculty of Medicine, Vasile Goldis Western University of Arad, 310025 Arad, Romania

**Keywords:** SARS-CoV-2, antibodies, COVID-19, blood donors, epidemiology, seroprevalence, Romania

## Abstract

Serological testing for SARS-CoV-2-specific antibodies, particularly those targeting the nucleocapsid protein, plays a key role in assessing past infection and estimating population-level seroprevalence. The seroprevalence of nucleocapsid antibodies against SARS-CoV-2 was evaluated in 1048 blood donors using the Elecsys Anti-SARS-CoV-2 electrochemiluminescence immunoassay. Participants completed a questionnaire to assess risk factors, symptoms during SARS-CoV-2 infection and vaccination status. The overall SARS-CoV-2 seroprevalence was 89.69%. Seroprevalence was not significantly associated with gender or age. In multivariate logistic regression, most investigated risk factors showed no significant association with seroprevalence. However, residence area and vaccination status were independently associated with SARS-CoV-2 seropositivity. Donors from rural areas had significantly higher odds of seropositivity (aOR = 1.68; 95% CI: 1.01–2.79; *p* = 0.045) compared to those from urban areas. Unvaccinated individuals were more likely to test positive for SARS-CoV-2 compared to vaccinated participants (aOR: 2.59; 95% CI: 1.35–4.99; *p* = 0.004). After three years of the COVID-19 pandemic, the prevalence of SARS-CoV-2 among blood donors was remarkably high, indicating that the vast majority of this population group had been exposed to the virus. This study highlights the risk factors for SARS-CoV-2 infection and the differences in antibody prevalence between vaccinated and unvaccinated individuals. Our findings underscore the pivotal role of vaccination in controlling the pandemic and provide valuable insights for policymakers in designing targeted strategies to curb future SARS-CoV-2 transmission.

## 1. Introduction

In late 2019, a new coronavirus, identified as Severe Acute Respiratory Syndrome Coronavirus 2 (SARS-CoV-2), surfaced in Wuhan, China, leading to an outbreak of atypical viral pneumonia. The new virus, causing Coronavirus Disease 2019 (COVID-19), rapidly spread globally due to its high transmissibility, generating the COVID-19 pandemic [1].

From 6 January 2025 to 2 February 2025, the global situation of SARS-CoV-2 revealed a 21% increase in new cases compared to the preceding 28-day interval, with more than 161,000 new cases reported. The number of new deaths increased by 14%, with 3300 new fatalities recorded. Up to 2 February 2025, over 776 million confirmed cases and more than seven million deaths have been reported worldwide. During this period, approximately 14,600 new hospitalizations and 1000 new intensive care unit (ICU) admissions were recorded, marking an increase of 10% in hospitalizations [2]. However, official reports do not accurately reflect the real degree of prevalence of SARS-CoV-2 infection in a population [3,4,5].

Blood donors’ screening has been used to monitor the population’s exposure to infections and indirectly to evaluate the herd immunity level in an exposed population. The identification of SARS-CoV-2 seroprevalence in blood donors can give an estimate of the community transmission of the virus infection [6].

Currently, limited data regarding the follow-up seroprevalence of SARS-CoV-2 antibodies in blood donors in the same region have been published in the medical literature. A study conducted in blood donors from England reported a SARS-CoV-2 seroprevalence of 4.4% in 2020 and 80.2% in 2022 [7]. Another study conducted in Australia revealed that SARS-CoV-2 nucleocapsid antibodies increased from 46.2% (from 9 June to 18 June 2022) [8] to 70.8% (from 29 November to 13 December 2022) [9]. Studies performed on blood donors from Western Romania have shown that SARS-CoV-2 seroprevalence increased rapidly from 1.51% in 2020 [3] to 41.04% in 2021 [10]. To our knowledge, no follow-up data regarding the prevalence of SARS-CoV-2 antibodies in blood donors is available since 2023. Therefore, the aim of the present study was to assess the prevalence of SARS-CoV-2 nucleocapsid antibodies in blood donors from Western Romania, the symptomatology and the potential risk factors associated with SARS-CoV-2 infection (including vaccination status) three years after the first seasonal evaluation of COVID-19.

## 2. Materials and Methods

### 2.1. Study Design and Population

The present study was conducted between 8 August and 2 September 2023 at the Regional Blood Transfusion Centre in Timisoara, Western Romania, and included 1048 blood donors. All participants were residents of Timis County (705,113 inhabitants) and complied with the donation eligibility criteria set by the Romanian Ministry of Health [11]. Study participants were grouped into five categories by their age at the time of sample collection: 18–29 years, 30–39 years, 40–49 years and 50–64 years.

### 2.2. Data and Questionnaire

Data analyzed in this study consisted of age, gender, area of residence, ABO blood groups, Rhesus antigen (Rh) and body mass index (BMI). The study participants filled out a questionnaire voluntarily. The whole process was conducted by physicians and specialized nurses under the strict supervision of the principal investigators.

The questionnaire contained questions regarding the potential risk factors for SARS-CoV-2 infection, such as cat(s) and/or dog(s) ownership, alcohol consumption, smoking (current or former smoker), educational level, income level, confirmed past SARS-CoV-2 infection by a positive polymerase chain reaction (PCR) test, number of SARS-CoV-2 infections and vaccination status. Additionally, the questionnaire included questions concerning the symptoms commonly associated with COVID-19, such as fever, cough, respiratory distress, chest pain, headache, anosmia (loss of smell), ageusia (loss of taste) and gastrointestinal symptoms.

### 2.3. Serologic Tests

Sera were tested at the Clinical Laboratory of the Municipal Clinical Emergency Teaching Hospital in Timisoara, a reference laboratory for COVID-19 testing in Romania. Samples were tested for SARS-CoV-2 antibodies using the Elecsys Anti-SARS-CoV-2 immunoassay, designed for Cobas e analyzers (Roche Diagnostics GmbH, Mannheim, Germany), which uses a recombinant nucleocapsid protein (N) for identifying the presence of the total antibodies against SARS-CoV-2 (IgM, IgA and IgG). Elecsys^®^ is a double-antigen sandwich assay that uses the nucleocapsid protein for specifically identifying the presence of the total anti-SARS-CoV-2 antibodies produced after infection, which are not generated after COVID-19 vaccination [12,13]. Antibodies to the nucleocapsid protein detect natural SARS-CoV-2 infection because this antigen is not targeted by the COVID-19 vaccines used in Europe at the time this study was conducted [13,14]. Elecsys^®^ has a specificity of 99.80% and a sensitivity of 99.5% for past infection in patients at ≥14 days after PCR confirmation. Interpretation of the test results was based on the manufacturer’s criteria: undetectable/non-reactive/negative if the cut-off index was <1.0 and detectable/reactive/positive if the cut-off index was ≥1.0 [15]. Quality control was performed according to the protocol specified by the manufacturer and the laboratory’s internal quality standards.

### 2.4. Statistical Analysis

Statistical analyses were performed using Stata 16.1 (StataCorp, College Station, TX, USA). Data were presented as numbers, percentages and mean ± standard deviation (SD). To investigate the association between positive cases and various variables, univariate logistic regression analysis was used. After identifying variables that were statistically significant (*p* < 0.05), multivariate logistic regression analysis was further conducted to identify the relevant risk factors.

In cases of missing values, we used listwise deletion, based on the assumption that the data were missing completely at random (MCAR). This approach was considered the default method for handling missing data in Stata [16].

We included all variables associated with the dependent variable and pre-defined variables that made clinical sense (Table 1, Table 2 and Table 3).

Crude odds ratios (cORs) were reported for univariate logistic regression, and adjusted odds ratios (aORs) were presented for multivariate logistic regression, both with their corresponding 95% confidence intervals (95% CI). Statistical significance for both logistic regression models was set at *p* < 0.05. Logistic regression analyses were performed following standard approaches used in epidemiological studies [17], though without the application of Directed Acyclic Graphs for covariate selection.

### 2.5. Ethics and Informed Consents

This study was approved (number E-4173/31.07.2023) by the Ethics Committee of the Municipal Clinical Emergency Teaching Hospital in Timisoara, Romania. Written informed consent was obtained from individuals who agreed to participate in this study.

## 3. Results

Of the 1048 study participants aged 18–64 years (mean age = 35.74 ± 10.33 years), 38.36% (402/1048) were females, and 72.23% (757/1048) were residents of the urban area.

The overall seroprevalence of SARS-CoV-2 nucleocapsid antibodies in blood donors from Western Romania was 89.69% (940/1048). A higher prevalence of detectable SARS-CoV-2 antibodies (92.78%; 270/291) was found in blood donors from rural areas compared to their urban counterparts (88.51%; 670/757). The cOR of 0.6 (95% CI: 0.36–0.98; *p* = 0.04) for urban residents revealed a significantly reduced probability of antibody detection rate compared to rural residents (Table 1).

**Table 1:** Seroprevalence of SARS-CoV-2 nucleocapsid antibodies in blood donors from Western Romania by age, gender, area of residence, ABO blood groups, Rhesus antigen and BMI groups.

Variables	Total Investigated *n* = 1048	*n* with Detectable SARS-CoV-2 N Antibodies (%)	cOR	95% CI	*p* Value
Age groups (years)					
18–29	328	301 (91.77)	Ref.		
30–39	363	326 (89.81)	0.79	0.47–1.33	0.38
40–49	232	205 (88.36)	0.68	0.39–1.19	0.18
50–64	125	108 (86.4)	0.57	0.3–1.09	0.09
Gender					
Female	402	365 (90.8)		Ref.	
Male	646	575 (89.01)	0.82	0.54–1.25	0.36
Area of residence					
Rural	291	270 (92.78)		Ref.	
Urban	757	670 (88.51)	0.6	0.36–0.98	**0.04**
ABO blood group					
O	416	374 (89.9)		Ref.	
A	410	372 (90.73)	1.10	0.69–1.74	0.69
B	160	140 (87.5)	0.79	0.45–1.39	0.41
AB	62	54 (87.1)	0.76	0.34–1.7	0
Rh					
Rh negative	165	151 (91.52)		Ref.	
Rh positive	883	789 (89.35)	0.78	0.43–1.4	0.4
BMI					
Underweight (<18.5)	8	8 (100%)	-		
Normal weight (18.5–24.9)	430	381 (88.6)	0.71	0.4–1.26	0.24
Overweight (25–29.9)	406	364 (89.66)	0.79	0.44–1.42	0.43
Obese (>30)	204	187 (91.67)		Ref.	
Total	1048	940 (89.69)			

*n* = number of blood donors, N = nucleocapsid, cOR = crude odds ratio, CI = confidence interval.

Non-smokers had a higher SARS-CoV-2 antibody prevalence of 91.95% (685/745), compared to the 84.11% (254/302) prevalence observed among smokers. The cOR for smokers was 0.46 (95% CI: 0.31–0.7; *p* < 0.001).

Blood donors with a previous confirmed diagnosis of COVID-19 had a higher seroprevalence of SARS-CoV-2 nucleocapsid antibodies (93.73%; 493/526) compared to blood donors who declared that they had never been infected with SARS-CoV-2 (85.77%; 446/520). The cOR was 2.48 (95% CI: 1.61–3.81; *p* < 0.001), suggesting that a confirmed infection with SARS-CoV-2 is associated with a higher prevalence of detectable antibodies.

Participants who had not been vaccinated had a higher prevalence of detectable antibodies of 94.79% (200/211), while vaccinated individuals had a prevalence of 88.45% (735/831). The cOR for vaccinated individuals having detectable antibodies was 0.42 (95% CI: 0.22–0.80; *p* = 0.008), showing a significantly lower prevalence of detectable antibodies post-vaccination (Table 2).

**Table 2:** Potential risk factors, vaccination status associated with detectable SARS-CoV-2 nucleocapsid antibodies in blood donors from Western Romania.

Potential Exposure to Risk Factors	Total	*n* with Detectable SARS-CoV-2 N Antibodies (%)	cOR	95% CI	*p* Value
Cat ownership	*n* = 1048				
No	757	684 (90.36)		Ref.	
Yes	291	256 (87.97)	0.78	0.51–1.2	0.26
Dog ownership	*n* = 1046				
No	679	606 (89.25)		Ref.	
Yes	367	332 (90.46)	1.14	0.75–1.75	0.54
Alcohol consumption	*n* = 1046				
No	356	325 (91.29)		Ref.	
Yes	690	613 (88.84)	0.76	0.49–1.18	0.22
Current smoker	*n* = 1047				
No	745	685 (91.95)		Ref.	
Yes	302	254 (84.11)	0.46	0.31–0.7	**<0.001**
Former smoker	*n* = 734				
No	482	446 (92.53)		Ref.	
Yes	252	227 (90.08)	0.73	0.43–1.25	0.26
Education level	*n* = 1043				
No formal education	1	1 (100)	-		
Primary education	5	5 (100)	-		
Gymnasium education	28	24 (85.71)	0.65	0.22–1.92	0.44
Vocational education	5	3 (60)	0.16	0.03–0.99	0.05
High school education	307	275 (89.58)	0.93	0.6–1.45	0.75
University or post-university	697	629 (90.24)		Ref.	
Income level in RON (EUR)	*n* = 967				
<1200 (240)	20	18 (90)		Ref.	
1200–2500 (240–500)	71	62 (87.32)	0.77	0.15–3.87	0.75
2500–5000 (500–1000)	360	324 (90%)	1	0.22–4.49	1
>5000 (>1000)	516	467 (90.5)	1.06	0.24–4.7	0.94
Confirmed past SARS-CoV-2 infection	*n* = 1046				
No	520	446 (85.77)		Ref.	
Yes	526	493 (93.73)	2.48	1.61–3.81	**<0.001**
No. of SARS-CoV-2 infections	*n* = 527				
0	2	2 (100)	-		
1	389	360 (92.54)	0.73	0.09–5.68	0.76
2	116	113 (97.41)	2.22	0.22–22.54	0.5
3	18	17 (94.44)		Ref.	
4	2	2 (100)	-		
Vaccination Status	*n* = 1042				
No	211	200 (94.79)		Ref.	
Yes	831	735 (88.45)	0.42	0.22–0.80	**0.008**

*n* = number of respondents per question, No. = number, Ref. = reference, cOR = crude odds ratio, CI = confidence interval, 1 EUR = 4. 97 RON (average of 2023).

A positive association was found between SARS-CoV-2 nucleocapsid antibodies seropositivity and blood donors who reported the following: (i) fever (94.34%, 300/318) (cOR: 2.34; 95% CI = 1.39–3.96; *p* = 0.001); (ii) cough (95.18%, 217/228) (cOR: 2.65; 95% CI: 1.39–5.03; *p* = 0.003); (iii) headache (93.95%, 233/248) (cOR: 2.04; 95% CI: 1.16–3.6; *p* = 0.013); (iv) anosmia (95.77%, 204/213) (cOR: 3.05; 95% CI: 1.51–6.14; *p* = 0.002); and (v) ageusia (96.45%, 190/197) (cOR: 3.66; 95% CI: 1.67–7.99; *p* = 0.001) (Table 3).

**Table 3:** Signs and symptoms associated with seroprevalence of SARS-CoV-2 nucleocapsid antibodies in blood donors from Western Romania.

Signs/Symptoms	*n*	*n* with Detectable SARS-CoV-2 N Antibodies (%)	cOR	95% CI	*p* Value
Fever					
No	730	640 (87.67)		Ref.	
Yes	318	300 (94.34)	2.34	1.39–3.96	**0.001**
Cough					
No	820	723 (88.17)		Ref.	
Yes	228	217 (95.18)	2.65	1.39–5.03	**0.003**
Respiratory Distress					
No	970	866 (89.28)		Ref.	
Yes	78	74 (94.87)	2.22	0.8–6.2	0.13
Chest Pain					
No	982	879 (89.51)		Ref.	
Yes	66	61 (92.42)	1.43	0.56–3.64	0.45
Headache					
No	800	707 (88.38)		Ref.	
Yes	248	233 (93.95)	2.04	1.16–3.6	**0.013**
Anosmia (Loss of Smell)					
No	835	736 (88.14)		Ref.	
Yes	213	204 (95.77)	3.05	1.51–6.14	**0.002**
Ageusia (Loss of Taste)					
No	851	750 (88.13)		Ref.	
Yes	197	190 (96.45)	3.66	1.67–7.99	**0.001**
Gastrointestinal Symptoms					
No	1006	903 (89.76)		Ref.	
Yes	42	37 (88.1)	0.84	0.32–2.2	0.73
Total	1048	940 (89.69)			

*n* = number of participants, cOR = crude odds ratio, CI = confidence interval.

Factors associated with SARS-CoV-2 seroprevalence in the univariate analysis were further assessed in a multivariate logistic regression analysis (Table 4). Blood donors residing in rural areas had a higher likelihood of antibody seropositivity (aOR: 1.68; 95% CI: 1.01–2.79; *p* = 0.045), indicating a 68% increase in the odds of testing positive for SARS-CoV-2 antibodies compared to urban residents.

Similarly, non-smokers showed a significantly increased probability of having detectable antibodies (aOR: 2.07; 95% CI: 1.36–3.16; *p* = 0.001), indicating a 107% increase in odds of testing positive for SARS-CoV-2 antibodies compared to smokers.

Unvaccinated individuals had a higher likelihood of antibody positivity (aOR: 2.59; 95% CI: 1.35–4.99; *p* = 0.004), indicating a more than twofold increase in odds of having detectable SARS-CoV-2 antibodies compared to those who were vaccinated.

## 4. Discussion

Results of the present study highlighted a high SARS-CoV-2 nucleocapsid antibody seroprevalence of 89.69% in blood donors from Western Romania. This result is higher than the seroprevalence of 47.7% reported by Offergeld et al. in blood donors from Germany in May 2022 [18] and the 80.2% seroprevalence reported by Harker et al. in their study conducted from June to November 2022 in Wales, United Kingdom [7].

**Figure 1:**
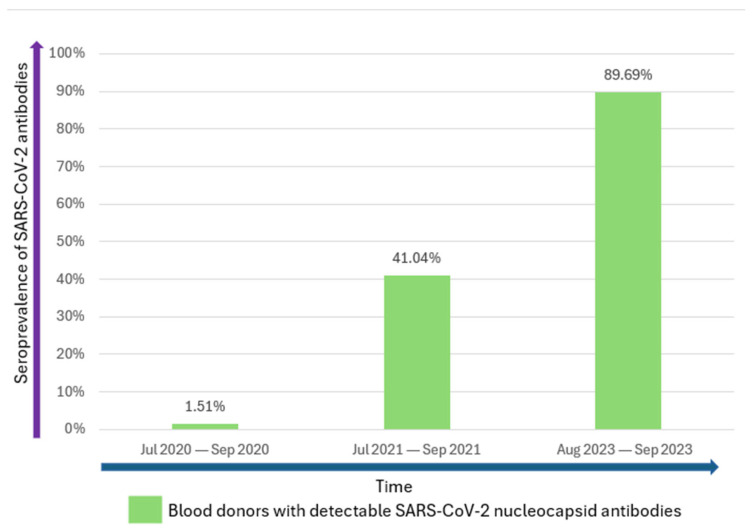
Seroprevalence of SARS-CoV-2 nucleocapsid antibodies in blood donors from Western Romania (2020–2023).

Previous studies conducted among blood donors in Western Romania have shown an increasing trend of SARS-CoV-2 seropositivity, from 1.51% seroprevalence of SARS-CoV-2 nucleocapsid antibodies observed between July and September 2020 [10] to 41.04% between July and September 2021 [3]. The 89.69% seroprevalence found in the present study showed a more than doubling value within the time span of two years (Figure 1).

A similar trend was also reported in a study conducted in Germany: nucleocapsid antibodies against SARS-CoV-2 in blood donors increased from 3.2% to 6.8% (January–April 2021) to 8.6% (September 2021) and to 47.7% (April–May 2022) [18]. In Wales, UK, serum samples collected from blood donors between June 2020 and November 2022 were tested for antibodies against SARS-CoV-2 nucleocapsid and showed that the seroprevalence increased from 4.4% (November 2020) to 16.7% (February 2021) and to 80.2% (November 2022) [7]. In Australia, the seroprevalence of SARS-CoV-2 nucleocapsid antibodies increased from 46.2% (9 June–18 June 2022) to 65.2% (23 August–2 September 2022) and to 70.8% (29 November–13 December 2022) [8,9].

In line with our results, several investigators reported little or inconsistent variation in SARS-CoV-2 seroprevalence by age or gender in blood donors [19,20,21]. In the Netherlands, a nationwide study conducted in donors found no differences after stratifying by age group or gender [20]. In Norway during winter/spring 2021, donor surveillance likewise showed no significant variation across age groups or gender [19]. In a recent large-scale surveillance study conducted in Austria, Siller and coworkers found a uniform seroprevalence across age and gender groups, without significant demographic differences [22]. Overall, the scientific literature suggests that age- or gender-related differences are small and context dependent, whereas regional transmission dynamics and timing within epidemic waves are the main drivers of seropositivity in blood-donor populations.

In our study of healthy blood donors, no differences in SARS-CoV-2 seroprevalence were observed across ABO or Rhesus blood groups. These results are consistent with our previous investigations in Romanian blood donors conducted both before and after the third pandemic wave [3,10]. Among donor cohorts, Quee et al. analyzed Dutch blood donors and did not identify ABO-specific differences in infection risk [23]. Reports from non-donor populations are mixed. An early hospital-based patient series in China, which included unhealthy inpatients, suggested a higher risk of SARS-CoV-2 infection in individuals with blood group A and a lower risk in those with group O [24,25]. A genome-wide association study, which screens the whole genome for links to disease, found that inherited differences related to ABO blood groups were associated with a higher likelihood of respiratory failure among hospitalized COVID-19 patients [26]. A large population-based administrative cohort from Ontario that included the general population with both healthy and unhealthy individuals found, at most, small effects [27]. A retrospective cohort of mass-gathering attendees in Spain, a largely healthy community sample, found no ABO differences in infection incidence [28]. A possible explanation for these conflicting findings is the health status of the participants. Recently, we examined adult outpatients in Western Romania and found that blood group A versus O was associated with higher SARS-CoV-2 seroprevalence only among participants with chronic diseases, but not in those without. This suggests that underlying health status may modify the relationship between the ABO blood group and the risk of infection [29].

The current research found that blood donors who smoke were less likely to have detectable SARS-CoV-2 nucleocapsid antibodies. Smokers may exhibit a lower antibody response against SARS-CoV-2 due to nicotine’s interaction with α7 nicotinic acetylcholine receptors on immune cells, which suppresses both innate and adaptive immune functions, impairing the development and activity of antibody-producing cells [30]. However, the relationship between smoking and susceptibility to SARS-CoV-2 infection remains debated. Some studies have reported a lower prevalence of infection among smokers, possibly reflecting underdiagnosis, reporting bias, or biological effects of nicotine on ACE2 receptor expression and immune modulation [31,32]. Conversely, other analyses have shown no protective association or even increased risk, particularly when considering disease severity and progression [33]. A recent pooled analysis from three Nordic countries found that tobacco use was not consistently associated with COVID-19 diagnoses, further highlighting the inconsistency of findings across populations [34]. Taken together, these data indicate that the observed lower antibody positivity among smokers in our cohort should be considered in context, as the direction and magnitude of the association may vary by population and methodological approach.

Vaccinated blood donors had a significantly lower seroprevalence of SARS-CoV-2 nucleocapsid antibodies when compared to unvaccinated ones. Our results are in accordance with Ferrari et al. (2023), who reported that vaccinated blood donors presented lower titers of SARS-CoV-2 nucleocapsid IgG antibodies [35]. In Romania, during 2021–2023, the COVID-19 vaccines in use were European Union-authorized spike-based products, such as Comirnaty (Pfizer-BioNTech, mRNA), Spikevax (Moderna, mRNA), Vaxzevria (AstraZeneca, ChAdOx1) and Jcovden/Ad26.COV2.S (Janssen), none of which included the nucleocapsid antigen [36]. Consistent with our findings, multiple blood-donor cohorts demonstrated protection associated with vaccination [37,38,39,40]. In a large longitudinal U.S. donor study, the incidence of primary infection was consistently lower in vaccinated blood donors compared to unvaccinated ones [39]. In the same donor surveillance network, higher spike-antibody concentrations, achieved through vaccination or hybrid immunity, correlated with reduced risk of first-time infection [37]. Quee et al. reported data from Dutch blood donors showing that booster vaccination produced strong antibody responses against SARS-CoV-2 and was associated with patterns consistent with enhanced protection in boosted individuals [40]. Canadian blood-donor surveillance showed widespread vaccine-induced anti-spike antibodies, followed by increases in infection-induced anti-N antibodies during Omicron, reflecting partial immune escape but continued benefit from vaccination [38]. Lastly, in our own Romanian outpatient cohort (Western Romania, Jan–Mar 2023), infection was most common in unvaccinated individuals and least common among those who received a booster, supporting that vaccination remained effective in this setting as well [29]. Regardless of the SARS-CoV-2 variant, vaccines also provide protection against COVID-19 hospitalization and COVID-19-associated emergency department/urgent care encounters [41].

In the present study, the most frequently reported symptoms were fever, cough, headache, anosmia and ageusia. While these findings are consistent with the results presented in previous descriptive studies [22,42,43], our multivariate analysis did not confirm a significant association between the investigated symptoms and seroprevalence. This suggests that anosmia and ageusia, which were prominent among symptomatic individuals, should not be interpreted as predictors of antibody positivity in this cohort. Previous research proposed that anosmia in COVID-19 may be linked to inflammatory processes in the nasal mucosa or to the virus affecting olfactory support cells via the ACE2 receptor, resulting in temporary loss of smell [44,45].

This study has several limitations. The high number of males, urban residents and university graduates may indicate a greater willingness or ability among these groups to participate in blood donation activities. This could be due to a variety of factors, including higher awareness levels and better access to donation centres [46]. In addition, we did not assess the presence of long COVID, and participants’ recall accuracy may contribute to a measurement error. Another limitation is that cross-sectional studies identify associations but not a temporal relationship, which complicates the determination of whether exposure preceded the observed outcome [47]. Our study may be subject to recall bias, as participants’ self-reported information could be influenced by imperfect memory or subjective interpretation [48]. We did not analyze different vaccine types separately but considered vaccination as a whole. Also, nucleocapsid antibody persistence is time-dependent, and waning may affect the accuracy of infection prevalence estimates, as shown with the Elecsys^®^ Anti-SARS-CoV-2 assays [23]. Lastly, blood donors do not represent the general population but, due to the high number of study participants, could offer insights into the current trends of SARS-CoV-2 infection among the general population.

## 5. Conclusions

After three years of the COVID-19 pandemic, the prevalence of SARS-CoV-2 infection among blood donors in Romania was remarkably high, indicating that the vast majority of this population group had been exposed to the virus. The present survey showed an increase in seroprevalence of more than twofold over a two-year period. This study highlights the risk factors for SARS-CoV-2 infection and the differences in antibody prevalence between vaccinated and unvaccinated individuals. Our findings underscore the role of vaccination in controlling the COVID-19 pandemic and provide valuable insights for policymakers in designing targeted strategies to curb future SARS-CoV-2 transmission, as most of the population has been exposed.

## Figures and Tables

**Table 4:** Factors associated with SARS-CoV-2 seroprevalence in blood donors from Western Romania (multivariate logistic regression analysis).

Risk Factor	aOR	95% CI	*p* Value
Area of residence	1.68	1.01–2.79	**0.045**
Current smoker	2.07	1.36–3.16	**0.001**
Confirmed past SARS-CoV-2 infection	1.65	0.84–3.21	0.14
Fever	1.20	0.56–0.59	0.64
Cough	1.47	0.66–3.27	0.35
Headaches	0.80	0.37–1.73	0.57
Anosmia	0.99	0.32–3.05	0.98
Ageusia	2.31	0.68–7.87	0.18
Vaccination status	2.59	1.35–4.99	**0.004**

aOR = adjusted odds ratio, CI = confidence interval.

## Data Availability

The original contributions presented in this study are included in the article. Further inquiries can be directed to the corresponding authors.

## Abbreviations

The following abbreviations are used in this manuscript:

COVID-19	Coronavirus Disease 2019
SARS-CoV-2	Severe Acute Respiratory Syndrome Coronavirus 2
